# Complete Plastid Genome Sequencing of Trochodendraceae Reveals a Significant Expansion of the Inverted Repeat and Suggests a Paleogene Divergence between the Two Extant Species

**DOI:** 10.1371/journal.pone.0060429

**Published:** 2013-04-05

**Authors:** Yan-xia Sun, Michael J. Moore, Ai-ping Meng, Pamela S. Soltis, Douglas E. Soltis, Jian-qiang Li, Heng-chang Wang

**Affiliations:** 1 Key Laboratory of Plant Germplasm Enhancement and Specialty Agriculture, Wuhan Botanical Garden, Chinese Academy of Sciences, Wuhan, Hubei, China; 2 University of Chinese Academy of Sciences, Beijing, China; 3 Department of Biology, Oberlin College, Oberlin, Ohio, United States of America; 4 Florida Museum of Natural History, University of Florida, Gainesville, Florida, United States of America; 5 Department of Biology, University of Florida, Gainesville, Florida, United States of America; J. Craig Venter Institute, United States of America

## Abstract

The early-diverging eudicot order Trochodendrales contains only two monospecific genera, *Tetracentron* and *Trochodendron*. Although an extensive fossil record indicates that the clade is perhaps 100 million years old and was widespread throughout the Northern Hemisphere during the Paleogene and Neogene, the two extant genera are both narrowly distributed in eastern Asia. Recent phylogenetic analyses strongly support a clade of Trochodendrales, Buxales, and *Gunneridae* (core eudicots), but complete plastome analyses do not resolve the relationships among these groups with strong support. However, plastid phylogenomic analyses have not included data for *Tetracentron*. To better resolve basal eudicot relationships and to clarify when the two extant genera of Trochodendrales diverged, we sequenced the complete plastid genome of *Tetracentron sinense* using Illumina technology. The *Tetracentron* and *Trochodendron* plastomes possess the typical gene content and arrangement that characterize most angiosperm plastid genomes, but both genomes have the same unusual ∼4 kb expansion of the inverted repeat region to include five genes (*rpl22, rps3, rpl16, rpl14,* and *rps8*) that are normally found in the large single-copy region. Maximum likelihood analyses of an 83-gene, 88 taxon angiosperm data set yield an identical tree topology as previous plastid-based trees, and moderately support the sister relationship between Buxaceae and Gunneridae. Molecular dating analyses suggest that *Tetracentron* and *Trochodendron* diverged between 44-30 million years ago, which is congruent with the fossil record of Trochodendrales and with previous estimates of the divergence time of these two taxa. We also characterize 154 simple sequence repeat loci from the *Tetracentron sinense* and *Trochodendron aralioides* plastomes that will be useful in future studies of population genetic structure for these relict species, both of which are of conservation concern.

## Introduction

The eudicot order Trochodendrales [1] contains only two extant genera, both of which are monotypic: *Trochodendron* Sieb. & Zucc. and *Tetracentron* Oliver. Historically, these two genera have been treated either as the separate families Trochodendraceae and Tetracentraceae, or as the combined family Trochodendraceae [1]–[7]. The Trochodendraceae *sensu* APG III [1] appear to have been widespread in the Northern Hemisphere during the Paleogene and Neogene [7]–[15]. However, the two extant species of the family have small geographic ranges and are restricted to eastern Asia [16]. *Trochodendron aralioides* Sieb. & Zucc. is a large, evergreen shrub or small tree native to the mountains of Japan to South Korea and Taiwan, and the Ryukyu Islands [2], [17], whereas *Tetracentron sinense* Oliver is a deciduous tree occurring in southwestern and central China and the eastern Himalayan regions. Both species are characterized by apetalous flowers arranged in cymose inflorescences and by loculicidal capsules that dehisce to release winged seeds [2], [5], [7], [18]. Although earlier researchers reported that wood of Trochodendrales wood lacked vessels and thus suggested that Trochodendrales were among the earliest-diverging angiosperms, recent research has documented the presence of vessels in the wood of both genera [2], [7], [19].

Molecular phylogenetic studies, including analyses of complete plastid genome sequences, have routinely recovered Trochodendrales as an early-diverging member of the clade *Eudicotyledoneae* (*sensu* [20]; all italicized clade names follow this system), specifically as part of a strongly supported clade with Buxales and *Gunneridae*, or core eudicots [21]–[27]. However, the relationships among Trochodendrales, Buxales, and *Gunneridae* have often been only weakly supported. In the 17-gene analysis of Soltis et al. [28], which included data from all three plant genomes, Trochodendrales and Buxales were subsequent sisters to *Gunneridae*, with 100% and 98% BS support, respectively. However, other studies have found Buxales to be sister to *Gunneridae* with only weak support [24], [26], [29]–[30], whereas in other analyses Trochodendrales have appeared as sister to *Gunneridae* [27], [31]–[32].

Complete plastid genome sequences have been used increasingly over the past decade to resolve deep-level phylogenetic relationships that have been unclear based on only a few genes. For example, recent plastid phylogenomic studies have helped to resolve key relationships among the earliest-diverging *Mesangiospermae* [33] as well as early-diverging *Eudicotyledoneae* and *Pentapetalae* [26], [34]. Indeed, the plastid genome represents an excellent source of characters for plant phylogenetics due to the generally strong conservation of plastid genome structure and its mix of sequence regions that vary tremendously in evolutionary rate [35]–[37], which enable plastid genome sequence data to be applied to phylogenetic problems at almost any taxonomic level in plants [26], [38]–[43]. It is now relatively inexpensive to generate complete plastid genome sequence due to rapid improvements in next-generation sequencing (NGS) technologies [25], [44]–[45] and due to the relatively small size of the plastid genome (∼150 kb) and its structural conservation, which enable dozens of plastomes to be multiplexed per sequencing lane and facilitate relatively straightforward genome assembly [45]–[48].

Despite the promise of NGS technology for plastid genomics, the complete plastomes of only eight genera of early-diverging eudicots have been reported: *Ranunculus* (Ranunculaceae, Ranunculales), *Megaleranthis* (Ranunculaceae, Ranunculales), *Nandina* (Berberidaceae, Ranunculales), *Nelumbo* (Nelumbonaceae, Proteales), *Platanus* (Platanaceae, Proteales), *Meliosma* (Sabiaceae, Sabiales), *Trochodendron* (Trochodendraceae, Trochodendrales) and *Buxus* (Buxaceae, Buxales). Previous phylogenetic analyses based on some of these complete genomes have not fully resolved the relationships among early-diverging eudicots, however; in addition to the uncertainty surrounding relationships of Buxales, Trochodendrales, and *Gunneridae*, the positions of Sabiales and Proteales remain poorly supported [26]–[27]. Plastome taxon sampling is still sparse in these clades, however, and additional sampling may help elucidate these recalcitrant relationships.

In addition to their important role in phylogenetics, plastid genomes may be rich sources of population-level data. The non-recombination and uniparental inheritance of most plastid genomes can make plastid genomes extremely useful for population genetics, particularly for tracing maternal lineages [49]–[50]. For example, chloroplast simple sequence repeats (cpSSR) have been widely used in plant population genetics [51], including within early-diverging eudicots, where numerous cpSSR loci have been reported from the plastid genome of the endangered species *Megaleranthis saniculifolia* (Ranunculaceae) [52].

Here we report the complete plastid genome sequences of *Tetracentron sinense* and *Trochodendron aralioides* (the protein-coding and rRNA genes of *Trochodendron* cp genome were used for phylogenetic analyses in Moore et al. [26], but the cp genome structure of this genus has never been reported), as well as the results of new phylogenetic analyses based on adding *Tetracentron* and *Megaleranthis* genomes [52] to the 83-gene data set of Moore et al. [26]. We also compare the plastid genome structure of *Trochodendron* and *Tetracentron*, including the characterization of a significant expansion of the inverted repeat in both taxa, and we estimate the divergence time between the two genera. Finally, we characterize the distribution and location of cpSSRs in both *Tetracentron sinense* and *Trochodendron aralioides*, which provided further opportunity to study the population genetic structures of these two ancient relict species.

## Results

### Sequencing and Genome Assembly

Illumina paired-end sequencing produced 892.11 Mb of data for *Tetracentron sinense*. We obtained 9912310 raw reads of 90 bp in length. The N50 of contigs was 13,981 bp and the summed length of contigs was 143,709 bp. The mean coverage of this genome was 5424.2×. After de novo and reference-guided assembly, we obtained a cp genome containing nine gaps. PCR and Sanger sequencing were used for filling the gaps. Four junction regions between IRs and SSC/LSC were first determined based on de novo contigs, and subsequently confirmed by PCR amplifications and Sanger sequencing, sequenced results were compared with the assembled genome directly and no mismatch or indel was observed, which validated the accuracy of our assembly. The genome sequences of *Tetracentron sinense* and *Trochodendron aralioides* have been submitted to GenBank (GenBank IDs: KC608752 and KC608753).

### General Features of the *Tetracentron* and *Trochodendron* Plastomes

**Figure 1:**
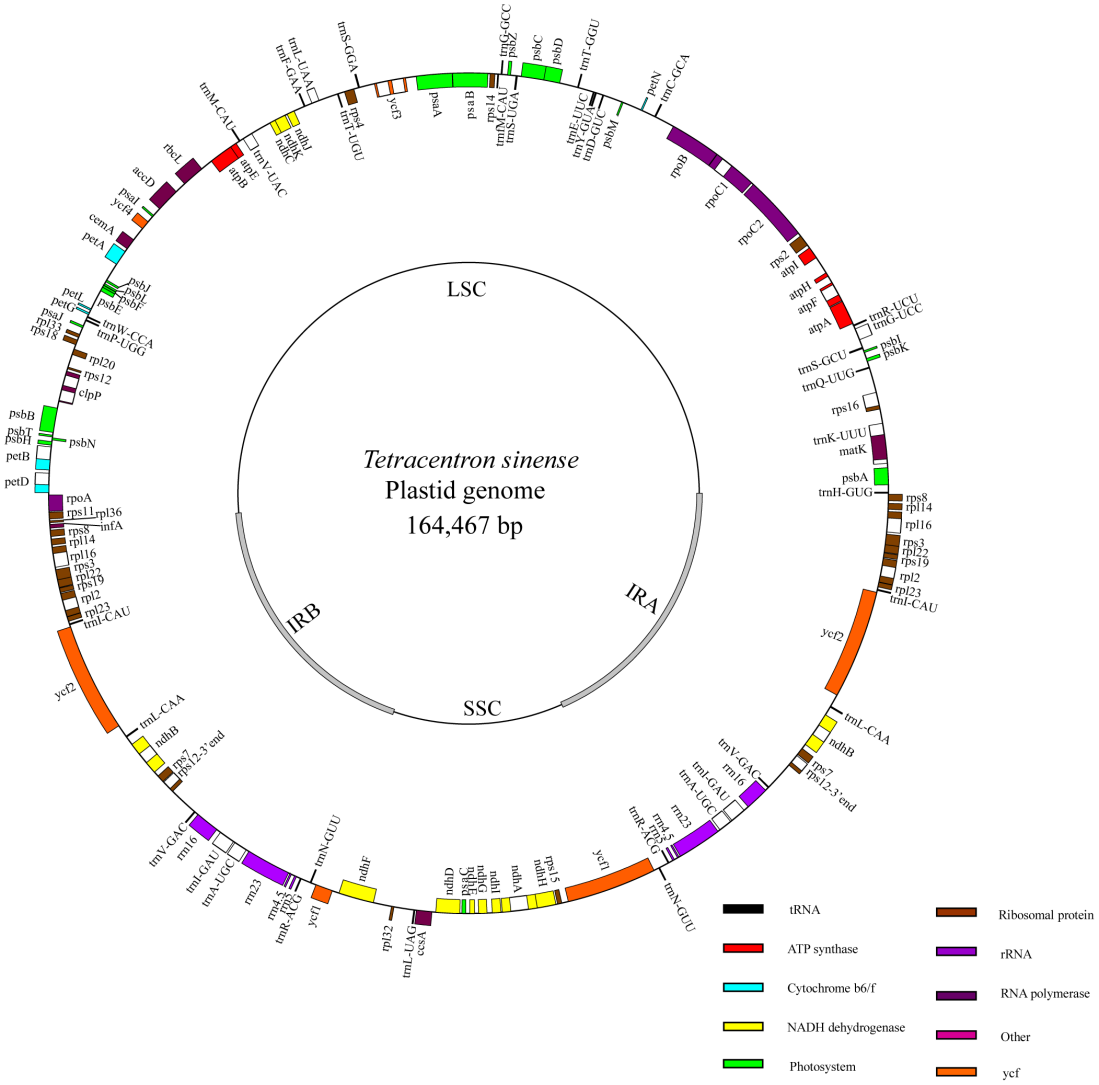
Map of the *Tetracentron sinense* plastid genome.

**Figure 2:**
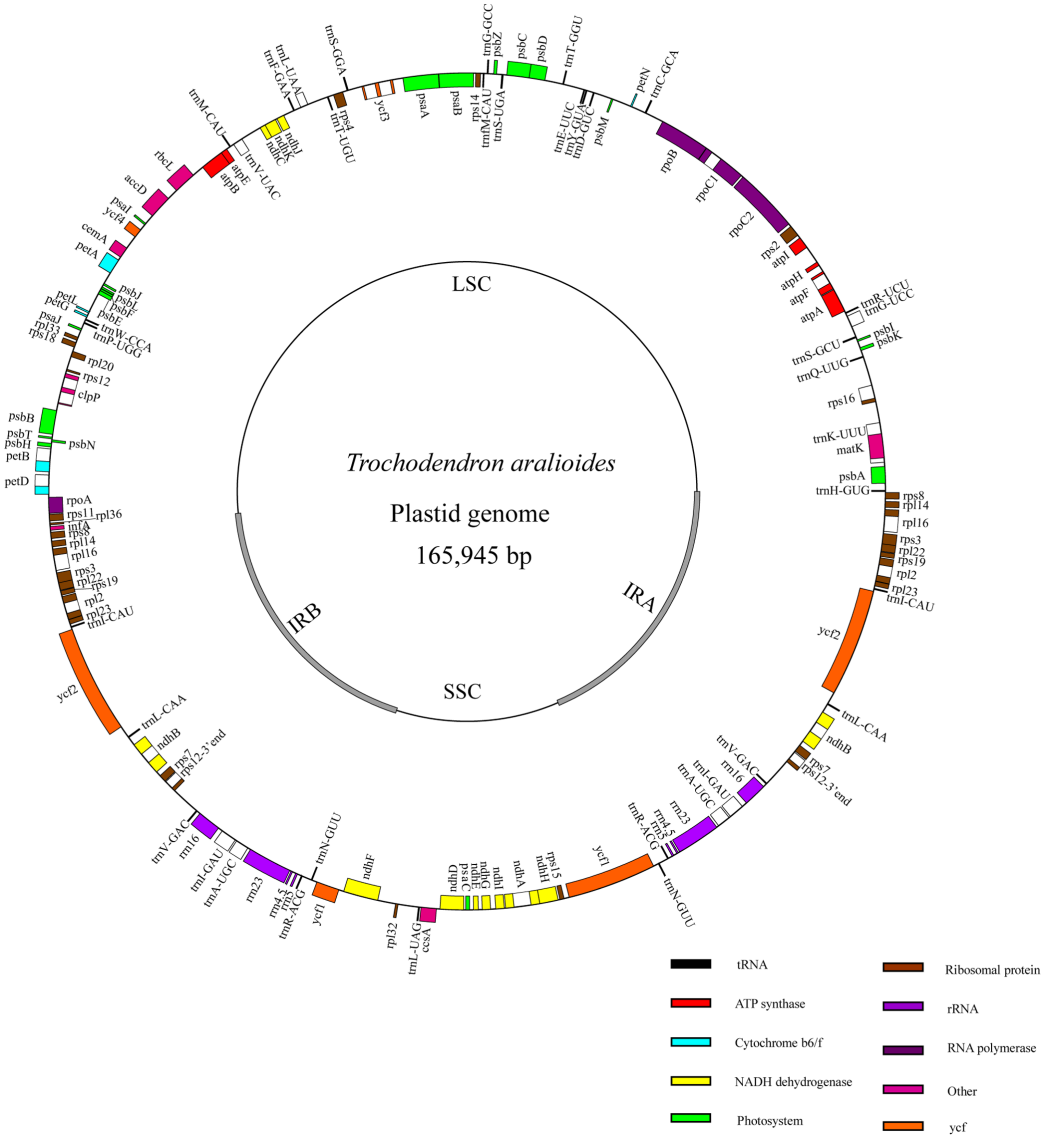
Map of the *Trochodendron aralioides* plastid genome.

**Figure 3:**
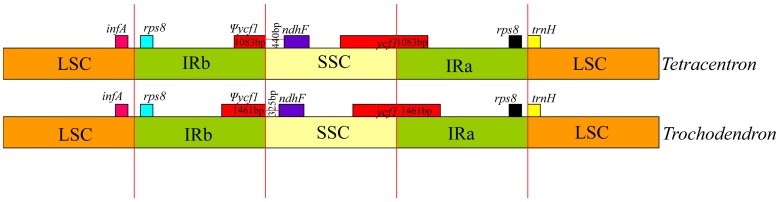
Comparison of the IR junctions in *Tetracentron* and *Trochodendron.*

The plastid genome size of *Tetracentron sinense* is 164,467 base pairs (bp) (Figure 1), and that of *Trochodendron aralioides* is 165,945 bp (Figure 2). Both genomes show typical quadripartite structure, consisting of two copies of an inverted repeat (IR) separated by the large single-copy (LSC) and small single-copy (SSC) regions (Table 1). The IR exhibits a significant expansion relative to most other angiosperms at the LSC/IR junction; specifically, the IR in both *Tetracentron* and *Trochodendron* has expanded to include the entirety of the *rps19, rpl22, rps3, rpl16, rpl14,* and *rps8* genes (Figures 1, 2). The SSC/IR boundary occurs within the *ycf1* gene, as is typical in angiosperms, but is slightly expanded in the *Trochodendron* genome to include 1461 bp of the 5′ end of *ycf1* (versus 1083 bp in *Tetracentron*; Figure 3). This expansion of the IR at the SSC junction contributes to the difference in length between the two Trochodendrales plastomes; the remainder of the difference is largely the result of length differences among various noncoding regions (Table 2).

**Table 1:** Basic characteristic of the *Tetracentron sinense* and *Trochodendron aralioides* plastid genomes.

	*Tetracentron*	*Trochodendron*
total genome length	164467	165945
IR length	30231	30744
SSC length	19539	18974
LSC length	84466	85483
total length of coding sequence	94699	95168
total length of noncoding sequence	69768	70777
overall G/C content	38.1%	38.0%

All values given are in base pairs (bp), unless otherwise noted.

**Table 2:** The principal noncoding regions contributing to the size difference between the *Tetracentron* and *Trochodendron* plastid genomes.

Spacer region or intron names	*Tetracentron*	*Trochodendron*	length difference
*trnK-UUU/rps16* spacer	870	1308	438
*rps16/trnQ-UUG* spacer	1529	1797	268
*trnS-GCU/trnG-UCC* spacer	505	658	153
*trnE-UUC/trnT-GGU* spacer	957	1316	359
*trnT-UGU/trnL-UAA* spacer	1199	1309	110
*petA/psbJ* spacer	1146	754	−392
*ycf1/ndhF* spacer	440	325	−115
*rpl16 intron	865	972	107

All sizes are in base pairs. The only locus residing in the IR is marked with an asterisk (*).

**Figure 4:**
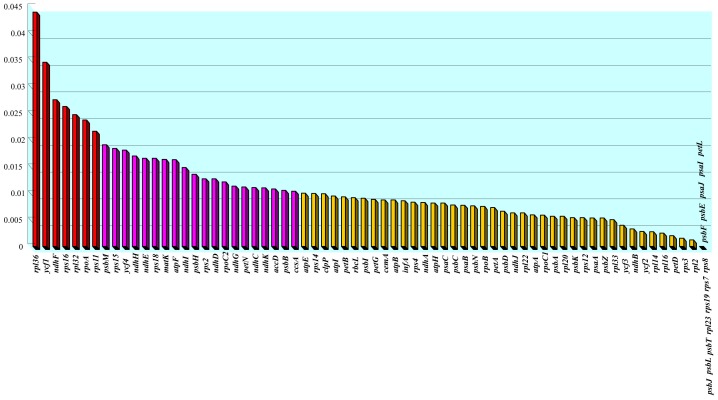
Amount of sequence divergence between the protein-coding genes of *Tetracentron* and *Trochodendron.*

**Figure 5:**
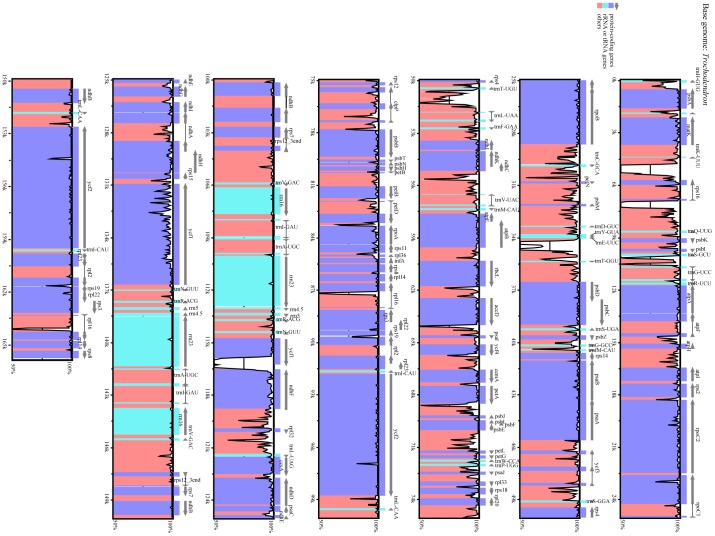
Sequence identity plot between *Trochodendron* and *Tetracentron*.

Both genomes contain 119 genes (79 protein-coding genes, 30 tRNA genes, and 4 rRNA genes) arranged in the same order, of which 24 are duplicated in the IR regions (Table 3). Sequence divergence between *Tetracentron* and *Trochodendron* in coding regions is low (Table 4, Figures 4, 5). Only 7 genes (*rps11*, *rpoA*, *rpl32*, *rps16*, *ndhF*, *ycf1*, and *rpl36*) exhibit divergences of more than 2%, and 12 genes have an identical sequence (Table 4, Figure 4). The genes *ndhF*, *ycf1*, and *rpl36* have the highest sequence divergences (2.7%, 3.5% and 4.4%, respectively). The coding regions account for 57.5% and 57.3% of the *Tetracentron* and *Trochodendron* plastid genomes, respectively. For both cp genomes, single introns are present in 18 genes, whereas three genes (*rps12, clpP,* and *ycf3*) have two introns (Table 5). The overall genomic G/C nucleotide composition is 38.1% and 38.0% for *Tetracentron* and *Trochodendron*, respectively; detailed A/T contents of different regions of the plastome for both genomes are listed in Table 6. Due to the lower A/T content of the four rRNA genes, the IR regions possess lower A/T content than the single-copy regions.

**Table 3:** List of genes present in the plastid genomes of *Tetracentron sinense* and *Trochodendron aralioides*.

	Group of genes	Name of genes
Protein synthesis and DNA replication	Ribosomal RNAs	*rrn4.5* (×2) *rrn5* (×2) *rrn16* (×2) *rrn23* (×2)
	Transfer RNAs	trnH-GUG trnK-UUU* trnQ-UUG trnS-GCU trnG-UCC* trnR-UCU trnC-GCA trnD-GUC trnY-GUA trnE-UUC trnT-GGU trnS-UGA trnG-GCC trnfM-CAU trnS-GGA trnT-UGU trnL-UAA*trnF-GAA trnV-UAC* trnM-CAU trnW-CCA trnP-UGG trnI-GAU* (×2) *trnL-CAA* (×2) *trnV-GAC* (×2) *trnI-GAU* (×2) trnA-UGC* (×2) *trnR-ACG* (×2) *trnN-GUU* (×2) *trnL-UAG*
	small subunit	*rps2 rps3 rps4 rps7* (×2) rps8 rps11 rps12* (×2) rps14 rps15 rps16* *rps18 rps19*
	Ribosomal proteins large subunit	rpl2* (×2) rpl14 rpl16* *rpl20 rpl22 rpl23* (×2) *rpl32 rpl33 rpl36*
	RNA polymerase	rpoA rpoB rpoC1* *rpoC2*
Photosynthesis	Photosystem I	*psaA psaB psaC psaI psaJ*
	Photosystem II	*psbA psbB psbC psbD psbE psbF psbH psbI psbJ psbK psbL psbM psbN psbT psbZ*
	Cytochrome b6/f	petA petB* petD* *petG petL petN*
	ATP synthase	atpA atpB atpE atpF* *atp*H *atp*I
	NADH dehydrogenase	ndhA* ndhB*(×2) *ndhC ndhD ndhE ndhF ndhG ndhH ndhI ndhJ ndhK*
	Large subunit of Rubisco	*rbcL*
Miscellaneous proteins	Subunit of Acetyl-CoA-carboxylase	*accD*
	c-type cytochrome synthesis gene	*ccsA*
	Envelope membrane protein	*cemA*
	Protease	clpP*
	Translational initiation factor	*infA*
	Maturase	*matK*
Genes of unknown function	Hypothetical conserved coding frame	*ycf1 ycf2*(×2) ycf3* *ycf*4

Genes with introns are marked with asterisks (*).

**Table 4:** Comparisons of the protein-coding genes of *Tetracentron* and *Trochodendron*.

Gene	Length in *Tetracentron*	Length in *Trochodendron*	Number of nucleotide differences	Proportion of nucleotide differences	Number of indel differences
*petL*	102	102	0	0	0
*psaI*	111	111	0	0	0
*psaJ*	129	129	0	0	0
*psbE*	252	252	0	0	0
*psbF*	120	120	0	0	0
*psbJ*	123	123	0	0	0
*psbL*	117	117	0	0	0
*psbT*	108	108	0	0	0
*rpl23*	288	288	0	0	0
*rps19*	279	279	0	0	0
*rps7*	468	468	0	0	0
*rps8*	399	399	0	0	0
*rpl2*	825	825	1	0.00121	0
*rps3*	657	657	1	0.00152	0
*petD*	504	504	1	0.00198	0
*rpl16*	501	501	1	0.00249	0
*rpl14*	369	369	1	0.00271	0
*ycf2*	6879	6897	19	0.00276	1
*ndhB*	1533	1533	5	0.00326	0
*ycf3*	507	507	2	0.00394	0
*rpl33*	201	201	1	0.00498	0
*psbZ*	189	189	1	0.00529	0
*psaA*	2253	2253	12	0.00533	0
*psbK*	186	186	1	0.00538	0
*rps12*	372	372	2	0.00538	0
*psbA*	1062	1062	6	0.00565	0
*rpl20*	354	354	2	0.00565	0
*rpoC1*	2049	2070	12	0.00586	1
*atpA*	1524	1524	9	0.00591	0
*rpl22*	486	480	3	0.00625	1
*ndhJ*	477	477	3	0.00629	0
*psbD*	1062	1062	7	0.00659	0
*petA*	963	963	7	0.00727	0
*rpoB*	3213	3213	24	0.00747	0
*psbN*	132	132	1	0.00758	0
*psaB*	2205	2205	17	0.00771	0
*psbC*	1422	1422	11	0.00774	0
*atpH*	246	246	2	0.00813	0
*psaC*	246	246	2	0.00813	0
*ndhA*	1095	1095	9	0.00822	0
*rps4*	606	606	5	0.00825	0
*infA*	234	234	2	0.00855	0
*atpB*	1497	1497	13	0.00868	0
*cemA*	690	690	6	0.0087	0
*petG*	114	114	1	0.00877	0
*psbI*	111	111	1	0.00901	0
*rbcL*	1428	1428	13	0.0091	0
*petB*	648	648	6	0.00926	0
*atpI*	744	744	7	0.00941	0
*clpP*	609	609	6	0.00985	0
*rps14*	303	303	3	0.0099	0
*atpE*	402	402	4	0.00995	0
*ccsA*	966	966	10	0.01035	0
*psbB*	1527	1527	16	0.01048	0
*accD*	1491	1491	16	0.01073	0
*ndhK*	822	858	9	0.01095	1
*ndhC*	363	363	4	0.01102	0
*petN*	90	90	1	0.01111	0
*ndhG*	531	531	6	0.0113	0
*rpoC2*	4137	4146	50	0.01209	1
*ndhD*	1503	1503	18	0.01264	0
*rps2*	711	711	9	0.01266	0
*psbH*	222	222	3	0.01351	0
*ndhI*	543	543	8	0.01473	0
*atpF*	555	555	9	0.01622	0
*matK*	1536	1536	25	0.01628	0
*ndhE*	306	303	5	0.0165	1
*rps18*	303	303	5	0.0165	0
*ndhH*	1182	1182	20	0.01692	0
*ycf4*	555	555	10	0.01805	0
*rps15*	273	273	5	0.01832	0
*psbM*	105	105	2	0.01905	0
*rps11*	417	417	9	0.02158	0
*rpoA*	1014	1014	24	0.02367	0
*rpl32*	162	162	4	0.02469	0
*rps16*	227	227	6	0.02622	0
*ndhF*	2223	2223	61	0.02744	0
*ycf1*	5688	5691	195	0.0345	6
*rpl36*	114	114	5	0.04386	0

Genes are ranked from lowest to highest proportion of nucleotide differences.

**Table 5:** Exon and intron lengths (bp) in plastid genes containing introns in *Tetracentron sinense* and *Trochodendron aralioides*, respectively.

Gene	Exon 1 (*Te/Tr*)	Intron 1 (*Te/Tr*)	Exon 2 (*Te/Tr*)	Intron 2 (*Te/Tr*)	Exon 3 (*Te/Tr*)
*trnK-UUU*	37/37		35/35		
*trnG-UCC*	24/24	698/698	48/48		
*trnL-UAA*	35/35	444/442	50/50		
*trnV-UAC*	39/39	583/585	37/37		
*trnI-GAU*	42/42	954/954	35/35		
*trnA-UGC*	38/38	794/794	35/35		
*petB*	6/6	793/797	642/642		
*petD*	8/8	704/709	496/496		
*atpF*	145/145	727/724	410/410		
*ndhA*	553/553	1106/1084	542/542		
*ndhB*	777/777	700/700	756/756		
*rpl2*	391/391	671/674	434/434		
*rpl16*	9/9	865/972	402/402		
*rps12*	114/114		232/232	538/536	26/26
*rpoC1*	432/432	728/714	1617/1638		
*clpP*	71/71	682/710	292/292	659/650	246/246
*ycf3*	124/124	734/725	230/230	731/758	153/153
*rps16*	40/40	831/844	227/227		

The *rps12* gene is trans-spliced, and hence the length of intron 1 is unknown.

**Table 6:** A/T content (%) of different regions in *Tetracentron* and *Trochodendron*.

Region	*Tetracentron*	*Trochodendron*
overall	61.86	61.98
LSC	63.50	63.74
IR	57.63	57.83
SSC	67.84	67.48
Protein-coding regions	61.58	61.53

### Characterization of SSR Loci

In all, 154 SSR loci (77 each from *Tetracentron sinense* and *Trochodendron aralioides*) were detected in the two plastid genomes, of which 123 are mononucleotide repeats, 28 are dinucleotide repeats, two are trinucleotide repeats, and one is a tetranucleotide repeat (Table 7). Nearly all of the SSR loci are composed of A/T repeats (Table 7), and these SSR loci are mostly present in noncoding regions. The tetranucleotide locus identified in *Tetracentron* is in the first intron of *ycf3*. The two trinucleotide loci in *Trochodendron* are both located in the spacer region between *trnK-UUU* and *rps16*. The unique C mononucleotide repeat from *Trochodendron* is present in the *trnV*-*ndhC* intergenic spacer region.

**Table 7:** Distribution of SSR loci in the plastid genomes of *Tetracentron* and *Trochodendron*.

Base	Length	Position in plastid genome
SSR loci in *Tetracentron*		
A	10	2085–2094 7164–7173 9478–9487 17266–17275 39220–39229 47812–47821 58880–58889 69930–69939 124816–124825 136417–136426 141648–141657
	11	9611–9621 46892–46902 47147–47157 50813–50823 75797–75807 80873–80883 82302–82312 133069–133079 160432–160442
	12	217–228 49977–49988 50332–50343 118899–118910 162450–162461 163452–163463 163940–163951
	14	65157–65170
	15	38842–38856
	17	39891–39907
	18	74838–74855
	22	72886–72907
T	10	5266–5275 6724–6733 9153–9162 19332–19341 54468–54477 63461–63470 67706–67715 107277–107286 112508–112517 117373–117382 118300–118309 121204–121213 126456–126465 130614–130623
	11	7004–7014 7679–7689 13144–13154 31361–31371 37925–37935 47779–47789 67810–67820 76013–76023 88492–88502
	12	55307–55318 71723–71734 84983–84994 85471–85482 86473–86484 118884–118895 119027–119038
	13	13902–13914
	14	72926–72939
AT	10	1734–1743 20833–20842 50404–50413–63181–63190
	12	4862–4873 12996–13007 114822–114833
	14	60686–60699
TA	10	34083–34092 34111–34120 114741–114750
	14	49132–49145
TAAA	20	46875–46894
SSR loci in *Trochodendron*		
A	10	118854–118863 126258–126267 142993–143002 163821–163830 18142–18151 40389–40398 41060– 41069 51091–51100 6136–6145 68969–68978 76681–76690 86529–86538
	11	134406–134416 16427–16437 30306–30316 39963–39973 51490–51500 70911–70921 81823–81833 9789–9799
	12	10420–10431 48058–48069 48322–48333
	13	164932–164944
	16	161805–161820 73777–73792 75726–75741
	15	46189–46203
	17	214–230 83299–83315 9304–9320
T	10	108427–108436 120424–120433 121028–121037 122665–122674 131951–131960 164891–164900 20189–20198 40375–40387 48933–4894253154–53163 53339–53348 5700–5709 6030–6039 68604–68613 72934–72943 83282–83291 87599–87608
	11	127885–127895 14709–14719 55604–55614 57547–57557
	12	50271–50282
	13	73814–73826 86485–86497
	14	76896–76909
	15	48889–48903
	16	89609–89624
AT	10	1724–1733 51556–51565 64459–64468
	12	4921–4932 4943–4954 4984–4995 4998–5009 5044–5055 5085–5096 5099–5110 5145–5156 5186–5197 5200–5211
	18	73275–73292
TA	10	1738–1747 21689–21698
TAA	18	5016–5033 5218–5235
C	10	55999–56008

### Phylogenetic and Molecular Dating Analyses

**Figure 6:**
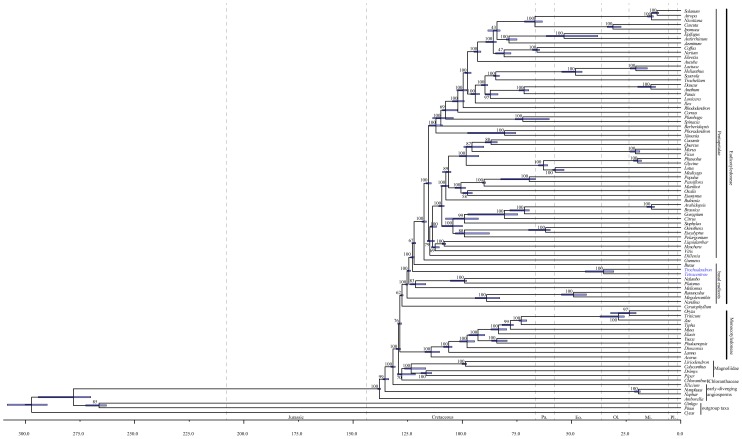
A maximum likelihood tree determined by GARLI (−ln *L* = −1095466.026) for the 83-gene, 88-taxon data set. Numbers associated with branches are ML bootstrap support values. Error bars around nodes correspond to 95% highest posterior distributions of divergence times based on 6 fossils using the program BEAST. Eo = Eocene, Mi = Miocene, Ol. = Oligocene, Pa = Paleocene, Pl = Pliocene.

ML analyses of the 83-gene, 88-taxon data set yielded a tree with a similar topology and bootstrap support (BS) values (Figure 6) as that of the plastid phylogenomic study of Moore et al. [26]. The clades of *Trochodendron*+*Tetracentron* and *Ranunculus*+*Megaleranthis* were supported with 100% ML BS support. Trochodendrales are sister to the remaining angiosperms with high support (BS = 100%), but Buxaceae are sister to Gunneridae with only 67% BS support.

Molecular dating analyses suggest that *Trochodendron* and *Tetracentron* diverged between 44-30 million ago. The crown group 95% highest posterior density (HPD) age estimates for other major lineages of *Pentapetalae* were as follows: *Superasteridae* (115-109 mya), Dilleniaceae+*Superrosidae* (116-112 mya), *Superrosidae* (114-111 mya), Santalales (98-75 mya), *Caryophyllales* (76-60 mya), *Asteridae* (104-99 mya), *Rosidae* (111-108 mya), Vitaceae+Saxifragales (114-110 mya), and Saxifragales (109-107 mya).

## Discussion

### Expansion of the IR Region in Trochodendrales Plastomes

The plastid genomes of *Tetracentron* and *Trochodendron* exhibit the typical gene content and genome structure of angiosperms [37], [53]–[54], with the notable exception of a significantly expanded IR region (Figures 1, 2, 3). This ∼4 kb expansion is responsible for the relatively large size of both Trochodendrales plastomes, which are ∼4–5 kb larger than the typical upper size range of angiosperm plastid genomes, including those of nearly all other early-diverging eudicots (Table 8). Significant expansion, contraction, and even loss of the IR appears to be an evolutionarily uncommon phenomena but are nonetheless associated with much of the more significant variation in plastome size in angiosperms. For example, the largest known angiosperm plastome, that of *Pelargonium* x *hortorum*, also possesses the largest known IR, at ∼76 kb in length [55]. Other significant IR expansions and contractions have been found in Campanulaceae [56]–[57], Apiaceae [58], and *Lemna* (Araceae) [59].

**Table 8:** Numbers of genes (including genes that span IR/SC junctions) in the IR regions of early-diverging eudicots.

Basal eudicot lineages	Species	Genes in IR region	cp genome size (bp)
Ranunculales	*Ranunculus macranthus*	20	155129
	*Megaleranthis saniculifolia*	19	159924
	*Nandina domestica*	19	156599
Proteales	*Nelumbo lutea*	18	163206
	*Platanus occidentalis*	19	161791
Sabiales	*Meliosma* aff. *cuneifolia*	18	160357
Buxales	*Buxus microphylla*	18	159010
Trochodendrales	*Tetracentron sinense*	24	164467
	*Trochodendron aralioides*	24	165945

### Impact of Additional Taxon Sampling on Basal Eudicot Phylogeny

The inclusion of *Megaleranthis* and *Tetracentron* in our analyses had no effect on the relationships among the major early-diverging eudicot lineages, and very little effect on support values. Of the basal splits among the eudicots with BS values less than 100% in both the current tree and that of Moore et al. [26], all were within 3% BS value. For example, the sister relationship of Buxales and *Gunneridae* is 70% in Moore et al. [26] vs. 67% with the inclusion of *Megaleranthis* and *Tetracentron*, and the sister relationship of Sabiales and Proteales has BS support of 80% in Moore et al. [26] vs. 83% in the current analyses. These similar values are unsurprising given that *Tetracentron* and *Trochodendron* are found to be relatively closely related in our analyses. Indeed, the relatively low sequence divergence between the *Tetracentron* and *Trochodendron* plastid genomes supports the taxonomic placement of Tetracentraceae within Trochodenraceae, as advocated by APG III [1]. Although it is possible that the addition of the noncoding regions of the plastid genome (or at least those noncoding regions that can be aligned) to our data set may improve support for these relationships, we may have to look to the other plant genomes for a confident resolution of relationships among the early-diverging eudicots. In fact, the sister relationship of Buxales and *Gunneridae* received high support (BS = 98%) in the 17-gene analyses of Soltis et al. [28], which employed a combination of 11 plastid genes, 18S and 26S nuclear rDNA, and 4 mitochondrial genes. However, the sister relationship of Sabiales and Proteales were more poorly supported (BS = 59%) in Soltis et al. [28].

### Divergence Time Between *Tetracentron* and *Trochodendron*

Cenozoic Trochodendrales fossils are known throughout the Northern Hemisphere, with the Paleocene *Nordenskioldia* the earliest certain fossil of the order [7]–[15]. Both *Tetracentron* and *Trochodendron* had wide distributions in the Northern Hemisphere during the Paleogene and Neogene. Fossil remains of *Tetracentron* have been found in Japan [60]–[61], Idaho [62], Princeton, British Columbia and Republic, Washington [63], and Iceland [15]; *Trochodendron* fossil remains have been reported from Kamchatka [64], Japan [11], Idaho and Oregon [11]–[12], Washington [7], and British Columbia [63]. Our estimate of the divergence time between the two genera of Trochodendraceae (44-30 mya) encompasses the recent estimate of 37-31 mya from Bell et al. [65], which was based on analysis of 567 taxa and three genes, as well as the mid-Eocene estimate of ∼45 mya derived from the *rbcL* analysis of Anderson et al. [66], which employed numerous fossil constraints from the early-diverging eudicots. The congruence among these studies and with the fossil record suggests that a mid- to late Eocene divergence for the two extant Trochodendraceae lineages may be a reasonable estimate.

### Analysis of Plastid SSR Loci in the Trochodendrales

Because microsatellite loci, including cpSSRs, often exhibit high variation within species, they are considered valuable molecular markers for population genetics [67]–[69]. A limited number of SSR loci were recently characterized for *Tetracentron* [70], but no cpSSR loci are available for Trochodendraceae. The 77 cpSSR loci that were identified in both *Tetracentron* and *Trochodendron* represent ∼42% more loci than the 54 loci reported in the plastid genome of *Megaleranthis* (Ranunculaceae), the only other early-diverging eudicot for which a comprehensive analysis of cpSSR loci is available. The abundant and varied cpSSR loci identified in Trochodendrales will be useful in characterizing the population genetics of both extant species, which are of conservation interest in the wild because of their relatively narrow, presumably relictual distributions, and decreasing numbers [71]. *Tetracentron* is officially afforded second-class protection in China.

## Materials and Methods

### Sample Preparation, Sequencing, and Assembly

Fresh leaves of *Tetracentron sinense* were collected from the Kunming Institute of Botany at the Chinese Academy of Sciences, and a voucher was deposited at the Herbarium of Wuhan Botanical Garden, Chinese Academy of Science (HIB). Chloroplast DNA was isolated following the protocol of Zhang et al. [45], and an Illumina library was constructed following the manufacturer’s protocol (Illumina). The DNA was indexed by tag and sequenced together with eight other species in one lane of an Illumina Genome Analyzer IIx at Beijing Genomics Institute (BGI) in Shenzhen, China. Illumina Pipeline 1.3.2 was used conducting image analysis and base calling. Raw sequence reads produced by Illumina paired-end sequencing were filtered for high quality reads which were subsequently assembled into contigs with a minimum length of 100 bp using SOAPdenovo [72] with the Kmer = 57. Contigs were aligned to the *Trochodendron aralioides* plastid genome using BLAST (http://blast.ncbi.nlm.nih.gov/), and aligned contigs were ordered according to the reference genome.

### Genome Annotation and Analysis

The *Tetracentron* and *Trochodendron* plastid genomes were annotated with DOGMA [73] and BLAST tools from NCBI (the National Center for Biotechnology Information). Physical maps were generated using GenomeVx [74] with subsequent manual editing. Sequence divergence between the *Tetracentron* and *Trochodendron* plastid genomes was evaluated using DnaSP version 5.10 [75], and genome sequence identity plots were generated using mVISTA [76] (http://genome.lbl.gov/vista/mvista/submit.shtml). Msatfinder ver. 1.6.8 [77] was used to identify SSR loci by manually setting repeat units.

### Phylogenetic and Divergence Time Analyses

All protein-coding sequences, as well as all rRNA sequences, were extracted from the *Tetracentron* and *Megaleranthis* plastome [52] and added manually to the 83-gene, 86-taxon alignment of Moore et al. [26]. ML analyses were performed on the concatenated 83-gene data set using the following partitioning strategy: (1) codon positions 1 and 2 together; (2) codon position 3; and (3) rRNA genes. The optimal nucleotide sequence model was selected for each partition using jModelTest 2.1.1 using the Decision Theory (DT) criterion [78]. The following models were selected: TVM+I+Γ for codon positions 1+2 and for codon position 3, and TIM1+ I+Γ for rRNA.

Partitioned ML analyses were conducted using GARLI 2.0 [79]. A total of ten search replicates were conducted to find the optimal tree, and nonparametric bootstrap support was assessed with 100 replicates [80]. All ML searches used random taxon addition to build starting trees.

Divergence times were estimated using BEAST version 1.7.4 [81], using the same dating strategies employed in Moore et al. [26]. In addition to the three calibration points (used in Moore et al. [26]) of minimum ages of 131.8 mya for angiosperms [82]–[85], 125 mya for eudicots [83], [86], and 85 mya for the most recent common ancestor of *Quercus* and *Cucumis* [26], we additionally constrained the stem lineage of Malpighiales using a minimum of 89.3 my [87] and the node uniting *Calycanthus* and *Liriodendron* using 98 my [88], and set the age of Proteales to a minimum of 98 my [89].
